# Enhancing Conductivity of Silver Nanowire Networks through Surface Engineering Using Bidentate Rigid Ligands

**DOI:** 10.1021/acsami.3c15207

**Published:** 2024-01-10

**Authors:** Wing Chung Liu, Joseph C. A. Prentice, Christopher E. Patrick, Andrew A. R. Watt

**Affiliations:** Department of Materials, University of Oxford, 16 Parks Road, Oxford OX1 3PH, United Kingdom

**Keywords:** silver nanowires, ligand exchange, conductive films, flexible electronics, molecular junctions

## Abstract

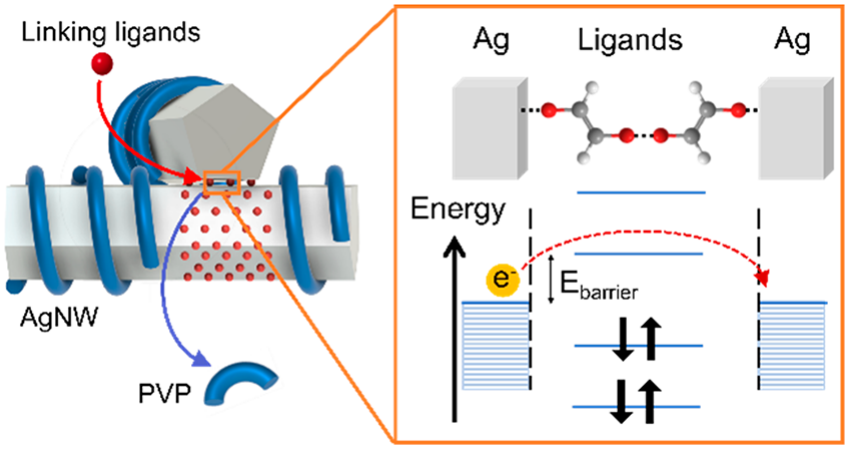

Solution processable metallic nanomaterials present a convenient way to fabricate conductive structures, which are necessary in all electronic devices. However, they tend to require post-treatments to remove the bulky ligands around them to achieve high conductivity. In this work, we present a method to formulate a post-treatment free conductive silver nanowire ink by controlling the type of ligands around the silver nanowires. We found that bidentate ligands with a rigid molecular structure were effective in improving the conductivity of the silver nanowire networks as they could maximize the number of linkages between neighboring nanowires. In addition, DFT calculations also revealed that ligands with good LUMO to silver energy alignment were more effective. Because of these reasons, fumaric acid was found to be the most effective ligand and achieved a large reduction in sheet resistance of 70% or higher depending on the nanowire network density. The concepts elucidated from this study would also be applicable to other solution processable nanomaterials systems such as quantum dots for photovoltaics or LEDs which also require good charge transport being neighboring nanoparticles.

## Introduction

The development of solution processable nanomaterials has created the possibility of fabricating large area electronic devices using high throughput and low cost techniques such as spin coating, blade casting, and various printing techniques.^1,2^ Their compatibility with low temperature processing and polymeric materials has also given rise to the rapidly expanding field of flexible electronics. Metallic nanomaterials such as silver nanowires (AgNWs) and nanoparticles are an important class of such materials. They are widely used to fabricate highly conductive structures which act as the electrodes and interconnects that are essential components in devices across many applications.^3^ In particular, AgNWs have garnered a lot of attention due to their unique properties. First, the high conductivity through the length of the AgNW allows conductive films to be made at relatively low metallic loading. At the same time, large pores are formed in a typical percolated AgNW network which give the film optical transparency, making them suitable for transparent electrodes in optoelectronics such as photovoltaics, light-emitting diodes, and touch screens.^4−8^ Moreover, their robust mechanical properties and high aspect ratio make them prime candidates for flexible and stretchable devices such as wearable sensors and energy devices.^9−13^

A key challenge in using metallic nanomaterials is the innately high junction resistance between adjacent nanoparticles. This is due to the presence of large surfactant ligand molecules surrounding the nanoparticles, which serves to improve processability by preventing their agglomeration in solution. In the case of AgNWs, polyvinylpyrrolidone (PVP) is a common polymer used as such a ligand. However, these ligands tend to impede charge transport once the nanomaterials are deposited onto devices. Common post-treatments such as thermal sintering, plasma treatment, or mechanical pressing are generally required to remove these ligands and fuse the nanowire junctions to improve the electrical properties of these metallic structures.^14−17^ However, such post-treatments may use up the thermal budget or alter the underlying materials in the device. Other sophisticated post-treatments using laser and microwave radiation have also been reported to perform the sintering while minimizing damage to the underlying materials.^18,19^ While these methods are effective in decreasing the junction resistance, they generally require additional equipment to be carried out. In addition, for printed structures which are typically denser, the sintering may cause large volume shrinkage in the printed structures and lead to rupturing and compromised mechanical properties.^20^ Chemical methods, such as ligand exchange, have also been demonstrated as effective post-treatment methods. These involve treating the metallic films with solutions containing smaller ligand molecules that can replace the original large ligands. With smaller ligands, the nanomaterials can achieve higher packing density and more efficient charge transfer.^21^ This is commonly reported in nanoparticle systems and has been applied in fabrication methods such as ligand-mediated layer-by-layer assembly.^22−24^ In some cases, the ligands can even induce sintering between nanoparticles, leading to dense and conductive films.^25^ While these methods can produce high quality conductive materials, ligand exchange as a post-treatment requires time for the exchange to chemically occur and additional washing steps to remove excess ligands, which decreases the process throughput.

A more effective way to circumvent these issues is to develop post-treatment-free inks.^20,26,27^ Current strategies to formulate post-treatment-free inks largely involve designing the ink composition to build in the ligand exchange process, which automatically occurs after the ink deposition step. Grouchko et al. demonstrated this by incorporating chloride ions into their silver nanoparticle ink. After the ink was printed on the substrate, the ligand exchange by the chloride ions was triggered by the increase in chloride concentration during ink drying.^27^ While this method was very effective in improving the charge transport between the nanoparticles leading to high conductivity films, the removed stabilizer ligands would inevitably still remain in the resultant device which may be unfavorable for its performance.

**Figure 1:**
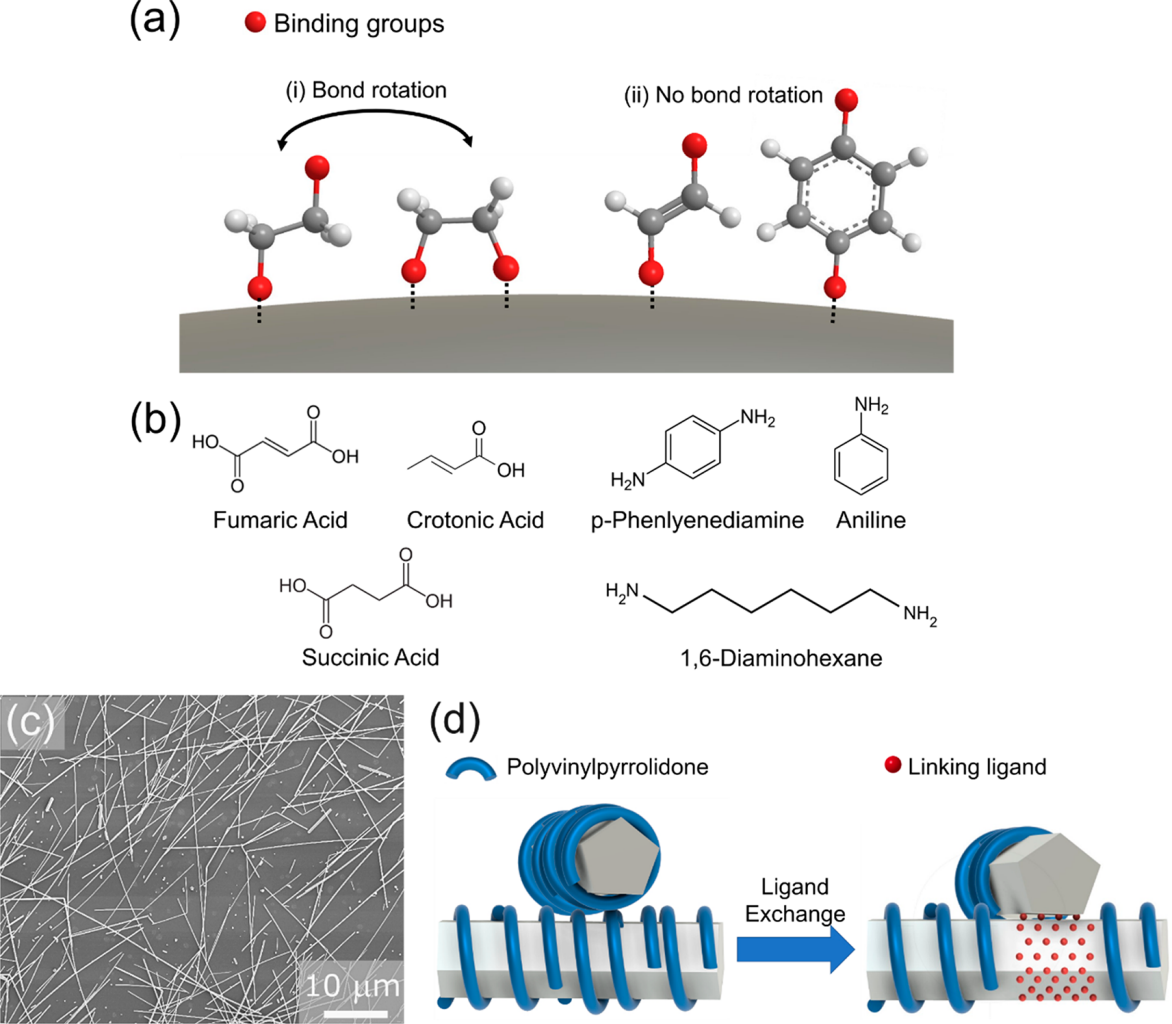
(a) Schematics of different ways bidentate ligands can bind onto a nanowire surface: (i) both binding groups on the ligand are bound to the same nanowire due to bond rotation in single bonds; (ii) unsaturated bonds in the molecule allow the ligand to remain rigid. (b) Acid and amine ligands used in this study. A mixture of both flexible and rigid monodentate and bidentate ligands is used. (c) SEM image of as-synthesized AgNWs used in this study. (d) Schematic diagram showing the potential mechanism for improvement in nanowire contact after ligand exchange.

In this study, we adopt the strategy of engineering the ligand shell on the AgNWs by using ligand exchange directly after their synthesis and prior to film fabrication. In contrast to existing methods, this strategy avoids the need for any post-treatments which simplifies the fabrication process, improves throughput, and minimizes any damage on underlying layers of materials. Because it has already been well established by prior reports that small ligand molecules are beneficial to improving charge transport, we aim to build on this and further elucidate the effect of the bond structure in small ligand molecules on the overall charge transport across AgNWs. Our key focus is on the use of bidentate ligands that have two binding functional groups, allowing them to act as linkers between nanomaterials to improve charge transport. In particular, we are interested in studying the effect of the rigidity of these bidentate ligands on the resultant charge transport between AgNWs. We posit that more flexible ligands with rotatable bonds can have both their functional groups bound onto a single AgNW (Figure 1a). This phenomenon has been reported in ligands adsorbed onto other nanomaterial systems.^28^ This configuration is undesirable, as their two groups should be bound to adjacent AgNWs for them to be effective linkers. Therefore, we hypothesize that rigid molecules with some degree of unsaturation in the form of C=C double bonds or aromatic rings should be present between the two binding functional groups. The results from our study will elucidate features that make ligands more effective in improving charge transport across nanomaterials and allow further ligand design to optimize the performance of solution processed nanomaterials.

## Results and Discussion

### Ligand Choice

To test our hypothesis, we used a mix of both saturated and unsaturated monodentate and bidentate ligands for comparison (Figure 1b). The use of carboxylic acids is the primary focus in this study as they are known to form coordinate bonds with silver surfaces and are generally nontoxic. Fumaric acid was used as the primary acid ligand of interest as it is the smallest dicarboxylic acid with a C=C double bond. Succinic acid and crotonic acid were therefore used as the flexible and monodentate comparison, respectively. To verify the general applicability of the study, amine ligands were also used to verify the hypothesis as they can also form coordinate bonds with silver surfaces. In this case *p*-phenylenediamine (PDA) was chosen as a bidentate ligand with a rigid aromatic ring. Aniline and 1,6-diaminohexane (DAH) were then used as the monodentate and flexible bidentate ligand, respectively, for comparison.

### Ligand Exchange Reaction

We first studied the ligand exchange reaction of these new ligands on the as-synthesized AgNWs. The AgNWs were synthesized through the polyol process using PVP as the capping agent.^29^ The resultant AgNWs had average lengths and diameters of approximately 20 μm and 80 nm, respectively (Figure 1c). Because both PVP and the new ligands are hydrophilic ligands, the ligand exchange was performed in a single solvent–ligand exchange reaction in methanol. It is generally difficult to achieve complete ligand exchange in single phase reactions unless the new ligand has a much stronger binding strength to the nanowire than the old ligand, which is not the case for this study.^30^ However, this may work to our advantage as complete ligand exchange in AgNWs would not be desirable either because the stabilizing ligands are still needed to maintain colloidal stability of the AgNWs in the ink.^31^ Therefore, the aim is to achieve a partial ligand exchange to have a mixture of stabilizing PVP ligands and charge transport boosting small ligands on the AgNWs to achieve a compromise between stability and conductivity (Figure 1c). Areas that undergo ligand exchange can come into closer contact with neighboring nanowires and improve electrical contact. The functional groups on the surface of the ligand shell can help to coordinate this linkage, as they can usually form strong hydrogen bonds with each other.

To facilitate the removal of PVP and exchange of new ligands, the AgNWs suspension was diluted before addition of a high concentration of new ligands. Varying amounts of ligands, in a desired molar ratio relative to the amount of silver denoted by *L*, were used in the ligand exchange.

Fourier transform infrared spectroscopy (FTIR) was first performed on dried ligand-exchanged AgNW (LE-AgNWs) to verify the presence of the new ligands (Figure S1). Table 1 summarizes some of the important distinguishing FTIR peaks observed that corresponded to the functional groups present in the ligands. Overall, the LE-AgNWs spectra generally showed some similar peaks that were present in the spectra of the pure ligands which suggests the presence of the new ligands on the LE-AgNWs. For the acid LE-AgNWs, peaks corresponding to the C–O stretch, C=O stretch, and O–H bend modes were observed in the spectra of all samples, showing the presence of carboxylic groups. =CH_2_ bending modes could also be observed in the fumaric acid and succinic acid, which gives further evidence that the ligands were successfully exchanged onto the AgNWs. Upon further comparison, it was found that the C–O and C=O stretch peaks were slightly shifted in all the acid LE-AgNW samples compared to the spectra of the pure ligands. This is likely due to a change in bond interactions in the carboxylic acid groups within the LE-AgNW samples compared to the pure ligands. The C–O peaks were consistently shifted to higher wavenumbers while the C=O peaks were shifted to lower wavenumbers. Overall, the peak separation between these two vibrational modes was more than 200 cm^–1^. According to the empirical rules presented in ref (32) (and references therein), this would imply that the oxygen on the C–O bond in each acid group was bound to one silver atom via coordinate bonds.^32^

**Table 1:** Peak Positions of Defining Functional Groups in the Pure Ligands and Their Corresponding LE-AgNWs

ligand	IR peaks from pure ligand (cm^–1^)	IR peaks from ligand exchanged nanowires (cm^–1^)	corresponding vibrational mode
fumaric acid	1006	995	=C–H bend
	1240	1266	C–O stretch
	1440	1409	O–H bend
	1683	1657	C=O stretch
succinic acid	1197	1226	C–O stretch
	1413	1397	O–H bend
	1685	1631	C=O stretch
crotonic acid	966	947	=C–H bend
	1220	1252	C–O stretch
	1425	1382	O–H bend
	1679	1646	C=O stretch
1,6-diaminohexane	1078	1087	C–N stretch
	1558	1525	N–H bend
*p*-phenylenediamine	1261	1238	C–N stretch
	1638	1614	N–H bend
aniline	1249	1230	C–N stretch
	1602	1588	N–H bend

In the amine LE-AgNWs, the presence of the C–N stretch and N–H bend vibrational peaks in the FTIR spectra showed the successful exchange of the amine ligands. The series of peaks within the region of 1400–1600 cm^–1^ in the aniline and PDA samples also corresponded to the vibrational modes from the benzene ring from the ligand as well. For the amine LE-AgNWs, the peak shifts were observed in both the C–N stretch and N–H bend peaks. In all samples, both the C–N stretch and N–H bend peaks were observed to decrease in wavenumber. Similar to the observations in the acid ligands, these shifts could indicate that the changing in bonding of the ligands in the LE-AgNWs samples through the amine group.

**Figure 2:**
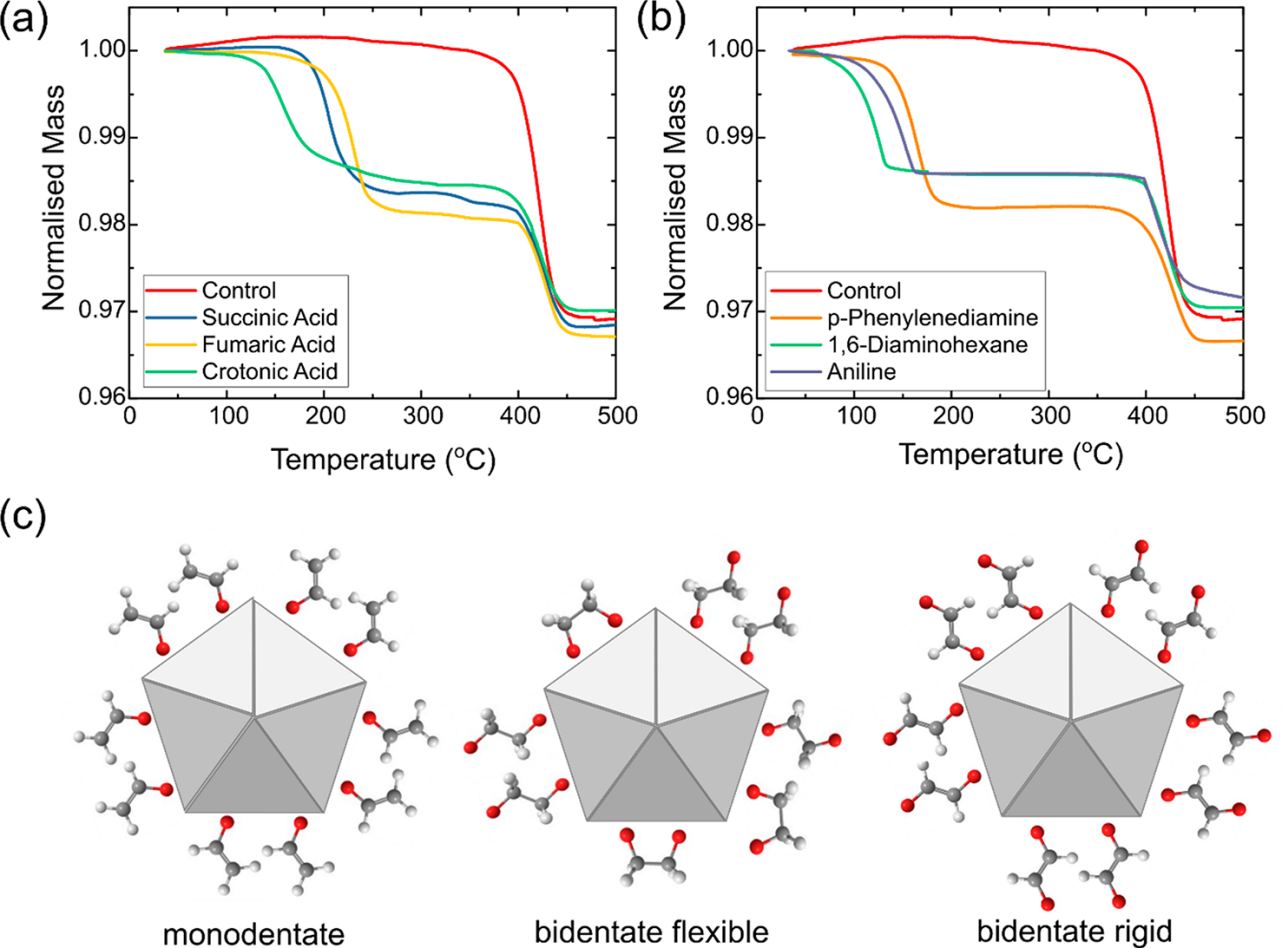
Normalized TGA curves for (a) acid ligand-exchanged AgNWs and (b) amine ligand-exchanged AgNWs. (c) Schematics of configuration of new ligands on nanowires without consideration of PVP for monodentate, bidentate flexible, and bidentate rigid ligands.

While the FTIR results do show the presence of the new ligands in the LE-AgNWs, it is unclear whether the new ligands actually replaced some of the pre-existing PVP or simply bound onto the existing PVP ligand shell. To distinguish between the two scenarios and obtain some quantification of the degree of ligand exchange, thermogravimetric analysis (TGA) was done on the different LE-AgNW samples (Figure 2). As different ligands generally decomposed at different temperatures, the weight loss observed in TGA at different temperatures would confirm that a different ligand was present.

The TGA curve for the control sample showed a single mass loss of 3.1% at 425 °C, corresponding to the decomposition of PVP on the AgNWs. These values are consistent with those reported in previous studies on PVP capped AgNWs of similar sizes as our AgNWs.^33^ For all of the other LE-AgNW samples, an additional mass loss was observed at a lower temperature, showing the presence of the new ligands within each sample. In general, the decomposition of the new ligands occurred at lower temperatures than PVP, which is expected as they are smaller organic molecules compared to PVP. The amount of mass loss corresponding to the new ligands was also different in different samples. At the same time, mass loss due to PVP was still observed, but the amount of mass loss was decreased. From the data, the relative molar ratios between the ligands and silver were calculated and are tabulated in Table 2. By comparison of the molar ratio of PVP to silver in each of the samples, it is clear that the amount of PVP present in the AgNW decreased after the ligand exchange reaction. This further proves the occurrence of ligand exchange instead of the simple addition of ligands onto the PVP.

**Table 2:** Decomposition Temperatures, Molar Ratios of New Ligands, and PVP to Silver in the Different LE-AgNWs

		molar ratio	
sample	decomposition temp (°C)	new ligand:Ag	PVP:Ag
control	425		0.0311
crotonic acid	161	0.0187	0.0142
fumaric acid	233	0.0178	0.0143
succinic acid	206	0.0143	0.0147
aniline	131	0.0159	0.0143
*p*-phenylenediamine	171	0.0164	0.0147
1,6-diaminohexane	159	0.0120	0.0149

Comparing between the results for the acid ligands, we observe that the amounts of crotonic and fumaric acid present in the LE-AgNWs were similar, while there is slightly less succinic acid being exchanged onto the nanowires. This observation is consistent with the proposed schematics in Figure 2c. Because each acid group binds via the monodentate mode, each crotonic acid molecule can bind to only one silver atom. At the same time, fumaric acid, despite having two acid groups, was effectively monodentate to a single nanowire due to its rigid structure. Therefore, similar amounts of crotonic and fumaric acids were exchanged onto the nanowires. Conversely, the flexible succinic acid allows bond rotation of the C–C bonds, causing them to behave as both monodentate and bidentate ligands on a single nanowire depending on factors such as availability of binding sites and their localized concentration in the solution. Because some of the succinic acid molecules can take up two binding sites on the silver surface, fewer molecules were exchanged onto the AgNWs as observed. Similar trends were also observed for the amine ligands. Similar amounts of monodentate aniline and rigid bidentate PDA were present on the LE-AgNWs, while less flexible bidentate DAH was present on the LE-AgNWs.

### Density of Available Linking Groups

Having verified the occurrence of the ligand exchange in the LE-AgNW samples, we proceeded to probe the density of available linking functional groups on the LE-AgNWs by measuring their zeta potentials in an aqueous medium. The zeta potential is an indication of the magnitude and polarity of the surface charge on the AgNWs which are largely dependent on the density and types of functional groups present in the ligand shell.^34,35^ AgNWs with more unbound functional groups on their surfaces should form links with each other more easily. At the same time, they should also show a greater change in the zeta potential after ligand exchange. The zeta potential signals can be further amplified by ionizing the carboxylic acid and amine groups linking group on the ligand shell to −COO^–^ and −NH_4_^+^, respectively, to generate more charge. To ensure that the acid and amine groups were fully ionized for fair comparison across ligands, the acid LE-AgNWs’ zeta potentials were measured at pH 8 while the amine LE-AgNWs’ zeta potentials were measured at pH 5.

**Figure 3:**
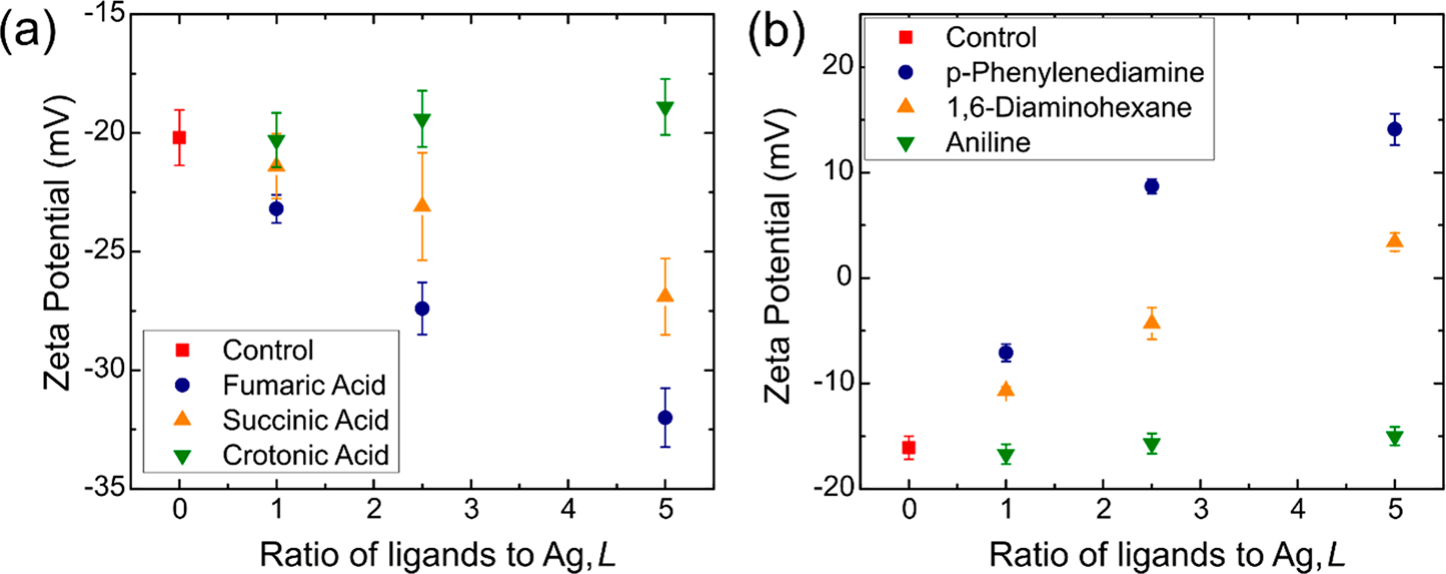
(a) Variation of the zeta potential of AgNWs when subjected to different degrees of ligand exchange using acid ligands and (b) amine ligands.

We measured the zeta potential of LE-AgNW samples after ligand exchange with varying ligand to Ag ratios, *L* (Figure 3). As expected, the acid LE-AgNWs have a more negative zeta potential than the control AgNWs due to having more negatively charged −COO^–^ groups. Conversely, the amine LE-AgNWs have a more positive zeta potential due to the −NH_4_^+^ groups. A higher *L* also caused a larger change in zeta potential, showing an increase in ligand exchange when more ligands are used during ligand exchange.

When the zeta potentials were compared among the acid ligands, the bidentate rigid fumaric acid resulted in the largest decrease in zeta potential followed by the bidentate flexible succinic acid, whereas the monodentate crotonic acid did not produce much change in zeta potential. Similarly for the amine LE-AgNWs, the bidentate rigid PDA showed the greatest increase in zeta potential, followed by the bidentate flexible DAH and last the monodentate aniline. These observations are consistent with our initial hypothesis about how the different types of ligands can attach to the AgNWs. The monodentate ligands do not create any charged binding groups on the LE-AgNW surface, whereas the rigid bidentate should create more charged groups compared to the flexible bidentate ligands as shown in Figure 2c. We note that the difference in zeta potential could also be caused by different amounts of ligands being exchanged onto the AgNWs as seen from the TGA results. However, we dismiss this as the main reason for the observed differences in zeta potential, as we estimate that the difference in amount of ligands on the AgNWs is insufficient to produce such a large change in zeta potentials (Supporting Information).

### Electrical Properties of Ligand Exchanged Nanowire Films

Next, we studied how the different densities of the linking groups on the AgNWs affect the overall electrical properties of the AgNW films. We used solvodynamic printing to fabricate the AgNW films as it can fabricate uniform films with good control of the areal density of AgNWs.^36^ This ensured that a similar number of nanowire junctions were formed in the different films, and therefore any differences in sheet resistance are attributed to the effect of the different ligands on the junction resistances. At the same time, the printing ink does not require many binders or viscosity modifiers, which results in highly conductive films.

**Figure 4:**
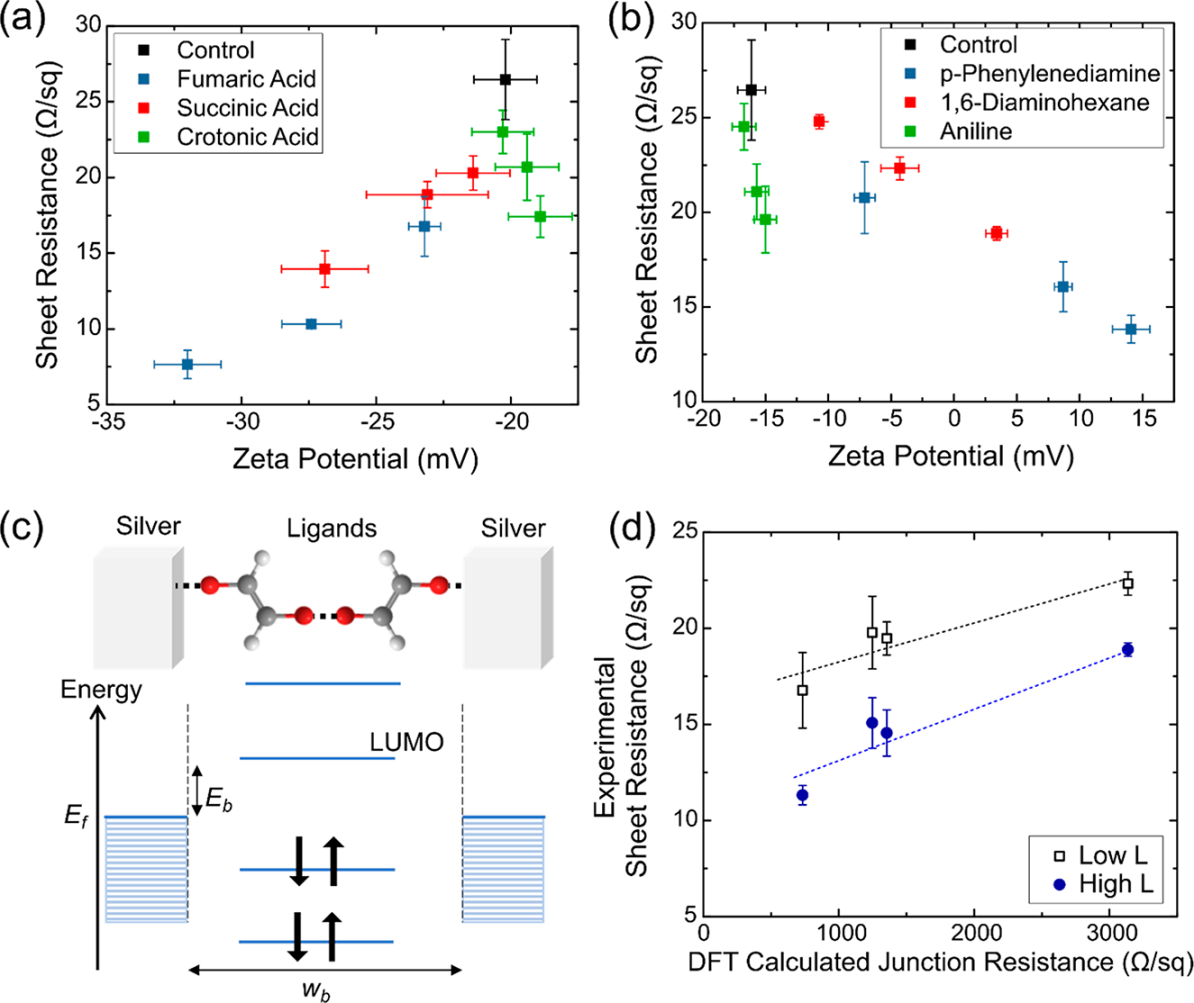
Variation in sheet resistances of AgNW films with zeta potential using different types of ligands for (a) acid ligands and (b) amine ligands. (c) Schematics of the energy band diagram in the AgNW–ligand–AgNW junction. *E*_f_ is the Fermi energy of silver, **E**_b_ is the energy barrier, and *w*_b_ is the barrier width for tunneling of electrons across the ligand. (d) Plot showing the relationship between the experimentally measured sheet resistance of LE-AgNW films using the four different bidentate ligands and their calculated tunneling junction resistances using DFT. The two set of data are tabulated from two sets of films with different densities of ligands.

The resultant variations of sheet resistances of the printed LE-AgNW films with their zeta potentials were measured (Figure 4a,b). As a baseline comparison, the films made using the control AgNWs achieved a sheet resistance of 26.5 Ω/sq. Conversely, all films made using acid or amine LE-AgNWs exhibited a lower sheet resistance than that of the control sample. In general, the monodentate ligand samples had sheet resistances higher than those of the bidentate ligand ones. This shows that bidentate ligands are more effective in improving the conductivity of AgNW films, likely due to their role as linkers. For the acid bidentate ligands, samples with a more negative zeta potential showed a greater degree of conductivity improvement. An equivalent observation can be made from the amine ligand samples, whereby the more positive zeta potential corresponded to a higher conductivity. This shows that a higher density of unbound linking groups improves the AgNW film conductivity. Because the conductivity through an individual AgNW was the same in each film, the increase in linking groups improved the junction resistance between AgNWs as hypothesized. Therefore, rigid ligands perform better because they can attain a higher density of linking groups on the AgNWs as indicated by zeta potentials with higher magnitudes.

While we have established that LE-AgNWs with a higher magnitude zeta potential correspond to more available linking groups and lower sheet resistances, there were still systematic differences in the sheet resistances between samples with different ligands even when they had similar zeta potentials. For example, the fumaric acid LE-AgNWs using *L* = 1 and 2 showed similar zeta potentials as the succinic acid LE-AgNWs using *L* = 2 and 3, respectively. This suggests that while they have similar density of linking groups, the fumaric acid LE-AgNW samples consistently have lower sheet resistances than the succinic acid. A similar observation can be made from the two bidentate amine LE-AgNWs. This observation suggests that the type of ligand directly affects the junction resistance between two adjacent nanowires.

To gain further insights into this observation, we quantified the charge transport through AgNW junctions across the different bidentate ligands. The nanowire junctions can be modeled as a metal–insulator–metal junction with the ligand as an insulator between two neighboring AgNWs (Figure 4c). Because of the short ligand lengths, tunneling should be the dominant charge transport mechanism across these junctions. Given the conditions of our conductivity measurements, we adopt the Fowler–Nordheim tunneling model which expresses the tunneling current, *I*, by the equation^37,38^
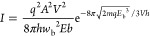
1where *q* is the electron charge, *A* is the junction area, *V* is applied voltage, *h* is Planck’s constant, *m* is the electron mass, *w*_b_ is the width of the tunneling barrier, and *E*_b_ is the magnitude of the energy barrier. Physically, the tunneling process is predominantly determined by the *E*_b_ and *w*_b_ formed by the ligand. According to the literature, the *E*_b_ can be approximated by the difference in energy level between the lowest unoccupied molecular orbital (LUMO) of the ligand molecule and the Fermi energy (*E*_f_) of the metal.^39^ Conversely, *w*_b_ is determined by the length of the ligand molecule and its conformation when adsorbed onto the AgNW surface. In order to obtain values for these quantities, we performed first-principles calculations based on density-functional theory (DFT),^40−42^ modeling the LE-AgNW junctions as ligands adsorbed via single functional groups on Ag-(001) slabs. We performed geometry relaxations to determine the equilibrium structure of the adsorbed ligands and thus obtain *w*_b_. We then extracted the energies of the ligand molecular orbitals with respect to the Ag Fermi energy and applied many-body and image charge corrections to the semilocal DFT values to obtain *E*_b_.^43−45^ Further details are provided in the Methods section, and the details related to the calculated *E*_b_ values are given in Table S2.

By inserting these calculated values into eq 1, the tunneling current, *I*, of each type of AgNW junction was obtained. Dividing *V* by calculated *I* gives the estimated junction resistances (Table 3). The results were consistent with the experimental results. Comparing between the two acid ligands, the junction formed with fumaric acid has the lower junction resistance. Therefore, the resultant LE-AgNW films have lower sheet resistances than the succinic acid LE-AgNW films even if the densities of linking groups are comparable. Similarly, the PDA ligand junction has a lower resistance than the DAH junction which leads to a lower film sheet resistance. We further analyzed the results by comparing all four bidentate ligands with each other. With the combination of the zeta potential and TGA results, we identified sets of LE-AgNWs samples with similar linking group densities (details in the Supporting Information) and plotted their film resistances against their junction resistances (Figure 4d). As expected, the ligands that cause a lower film sheet resistance are also predicted by DFT to form more conductive junctions. A roughly linear trend was observed between the junction resistance and film sheet resistance, which matches the results reported in the current literature as well.^46,47^

**Table 3:** Energy Barrier Height and Width Obtained from DFT Calculations and the Estimated Tunneling Junction Resistance in Each Sample

ligand	energy barrier (eV)	barrier width (Å)	junction resistance (Ω)
fumaric acid	2.61	16.9	733
succinic acid	4.44	17.6	1354
*p*-phenylenediamine	4.19	17.4	1248
1,6-diaminohexane	4.50	26.6	3136

Overall, fumaric acid forms the nanowire junction with the lowest resistance due to a combination of its small molecular size and the close proximity of its LUMO to the silver Fermi energy. This resulted in an overall decrease in sheet resistance to 7.66 Ω/□ for the *L* = 5 sample, corresponding to a reduction in sheet resistance by approximately 70%. This is a good result, as such a large conductivity improvement can be achieved without the use of post-treatments.

**Figure 5:**
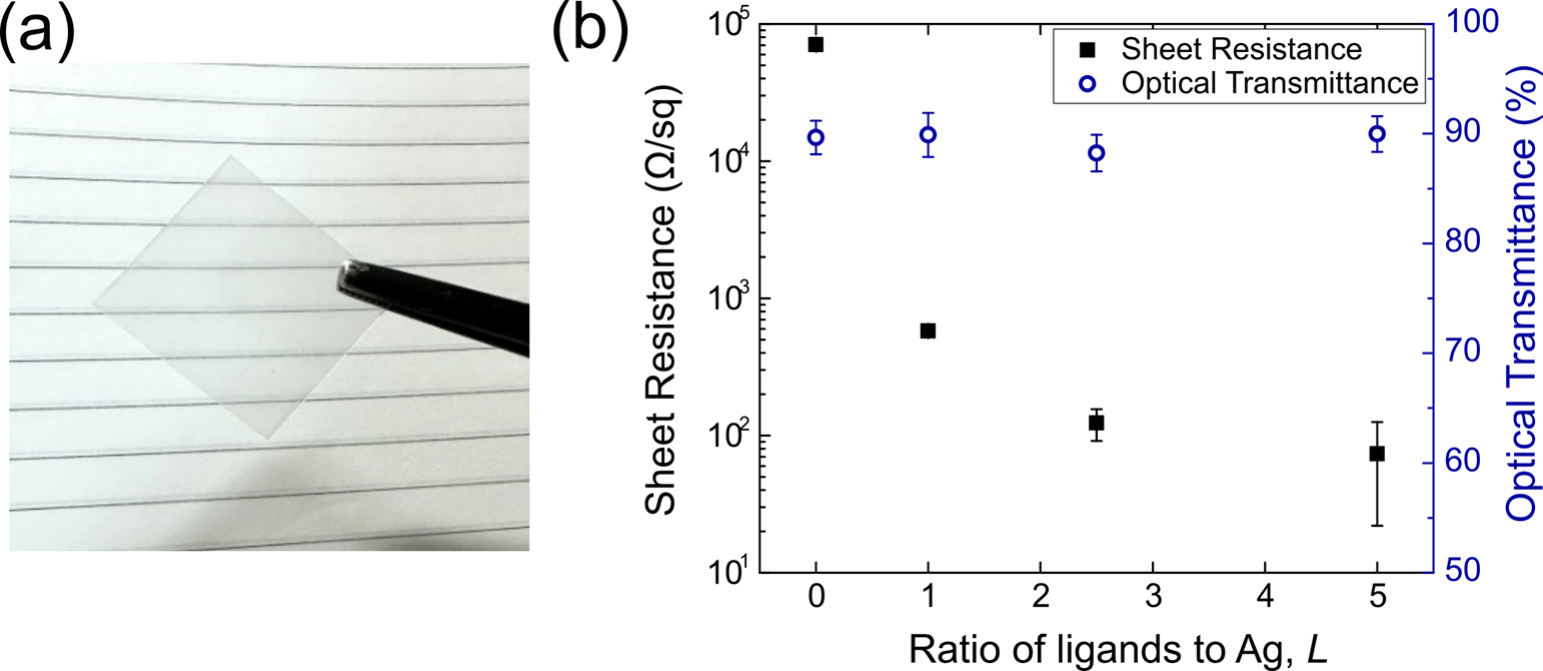
(a) Optical image of a piece of glass coated with ligand exchanged AgNWs. (b) Variation in sheet resistance and optical transmittance of spin coated AgNW films using fumaric acid LE-AgNWs.

The reduction in the sheet resistance can be even greater in films with lower AgNW densities. The overall resistance is a combination of junction resistance between neighboring nanowires and the resistance through individual AgNWs. In dense films, many parallel paths exist in the film and reduce the effective junction resistance within the film. Conversely, in films with lower AgNW densities, fewer parallel conduction paths lead to a high contribution of junction resistance to the overall film resistance. We demonstrate this by fabricating transparent conducting films, a common application of AgNWs, with fumaric acid LE-AgNWs using spin coating (Figure 5a,b). All samples had similar optical transmittances of around 89%, which indicates that fumaric acid did not affect the final nanowire loading on the spin coated samples. The film made using the as-grown AgNWs showed a high sheet resistance of approximately 70 kΩ/□ due to the inherently high junction resistance coupled with low AgNW density. Conversely, as more fumaric acid is exchanged onto the AgNWs, the junction resistance is reduced, leading to a decreased sheet resistance of 73.7 Ω/□. This corresponded to a decrease by 3 orders of magnitude due to the large contribution of junction resistance in such low loading films. One potential way to further reduce the sheet resistance is to use computational methods to identify other small bidentate ligands with more optimal alignment between their LUMO energy level and the silver Fermi energy. This will further decrease the tunneling barrier and the junction resistance between the nanowires.

## Conclusion

In conclusion, we have demonstrated a method to formulate a conductive AgNW ink capable of forming highly conductive films without the need for any post-treatment. This was made possible by partial ligand exchange of PVP with smaller bidentate rigid ligands. These ligands act as linking molecules between neighboring nanowires and reduce the resultant junction resistance and overall sheet resistance. It was found that the bidentate ligands with a rigid molecular structure were more effective linkers, as they could maximize the number of available binding groups on the ligand shell for forming linkages. Among all of the ligands that were used, fumaric acid showed the best performance for conductivity improvement by 70% or higher depending on the nanowire density. Aside from being a bidentate rigid ligand, fumaric acid also has a small size and good LUMO band alignment with the silver Fermi energy to allow easy tunneling of electrons across the AgNW junction. Overall, the concepts elucidated in this study would also be applicable in other nanomaterial systems such as quantum dots for electronic applications, in which charge transport between nanoparticles is important. Further fundamental studies on the relation between ligand structure and energy level can also be done to identify more effective ligands to further improve the current results.

## Materials and Methods

### Materials

Silver nitrate, ethylene glycol (EG), copper(II) chloride dihydrate, polyvinylpyrrolidone (PVP) with average molecular weight of 55000, fumaric acid, succinic acid, crotonic acid, *p*-phenylenediamine, 1,6-diaminohexane, aniline, and hydrochloric acid were purchased from Sigma-Aldrich and used without modification.

### Synthesis of AgNWs

In a typical synthesis, 0.18 g of silver nitrate was first dissolved in 1.5 mL of ethylene glycol. In a separate conical flask, 0.8 g of PVP was dissolved in 20 mL of ethylene glycol and heated to 150 °C in an oil bath with magnetic stirring. 20 μL of a 0.15 M aqueous copper(II) chloride solution was then added to the PVP solution. Once the solution was mixed homogeneously, the silver nitrate solution was added, and the reaction mixture was left under heating and magnetic stirring for 1 h. After the reaction, the nanowires were washed with methanol and subsequently centrifuged at 2500 rpm for 10 min. This was repeated 3 times before finally redispersing the nanowires into methanol to form a suspension.

### Ligand Exchange of Silver Nanowires

To facilitate the ligand exchange reaction, the methanolic AgNW suspension was first diluted to 1 mg/mL, and the new ligands were added into the suspension. The ligands were then added to the suspension based on a specific molar ratio with respect to the silver, denoted by *L*. For each type of ligand, an amount corresponding to *L* = 1, 2.5, and 5 was added to the AgNW suspensions. The resultant mixtures were magnetically stirred overnight for 24 h. The suspensions were then centrifuged at 2500 rpm for 10 min. The supernatant was discarded, and the remaining sediment was redispersed in different solvents to form suspensions for different uses as described subsequently.

### Fabrication of Silver Nanowire Films

AgNW films were fabricated by using spin coating and solvodynamic printing. Glass substrates were first cleaned by ultrasonication in decon soap solution, DI water, acetone, and isopropanol sequentially at 40 °C for 5 min each.

The spin coating ink was prepared by dispersing the ligand exchanged AgNWs (LE-AgNWs) into methanol at a concentration of 1 mg/mL. A glass substrate was placed in a spin coater (Ossila) and spun at 1000 rpm, while 1 mL of ink was deposited onto the spinning substrate dropwise using a micropipet. The AgNW-coated glass substrate was left to spin for 1 min to allow the ink to dry completely.

The printing ink was prepared by dispersing the LE-AgNWs in a solution of deionized (DI) water with 15 wt % EG with a AgNW concentration of 6 mg/mL. Hexane was used as the carrier solvent. The nominal ink and carrier solvent flow rates were set at 10 and 20 μL/min, respectively. The substrate velocity was set at 5 mm/s. A glass substrate was placed onto an *x*–*y* stage below the printhead. The stage movement was programmed to print a AgNW film on the glass substrate. Three layers were printed for each film. The AgNW films were dried on a hot plate at 80 °C for 5 min after printing each layer.

### First-Principles Calculations

Calculations were performed using a generalized-gradient approximation to DFT (Perdew–Burke–Ernzerhof, PBE functional),^41^ accounting for van der Waals interactions within the Tkatchenko–Scheffler scheme.^42^ The DFT equations were solved using plane-wave basis sets, periodic boundary conditions, and on-the-fly generated norm-conserving pseudopotentials, as implemented in the CASTEP software package.^40^ A 750 eV cutoff energy was used for the plane-wave expansions. The models of the LE-AgNWs were constructed by optimizing the structure of fcc Ag, sampling reciprocal space on a high density (0.01 Å^–1^) grid. The obtained lattice constant (4.148 Å) was then used to construct a 2 × 2 Ag-(001) slab, consisting of five Ag layers separated by 20 Å of vacuum. For each of the six ligands listed in Figure 1b, an interface model was constructed by positioning the ligand on the slab in a monodentate configuration and performing a geometry optimization until the force on each atom was below 1 meVÅ^–1^. Both the lateral cell dimensions and the lowest three Ag layers were kept fixed in the optimization in order to mimic the larger Ag nanowire. To obtain the equilibrium slab–slab distance *w*_b_ from these relaxed structures, we extracted the molecule only, added a replica of the molecule reflected in the *z*-direction (as if attached to another slab), and calculated the energy of this molecular dimer system as the distance between the molecules in the *z*-direction was varied. The separation with the smallest energy was then added to 2 times the height of the molecule (calculated as the difference between the largest atomic *z*-coordinate and the average *z*-coordinate of the Ag atoms at the surface) to give *w*_b_. In order to calculate the quantity *E*_b_ (approximated as the difference between the ligand LUMO and Ag Fermi energy, *E*_F_), we plotted the atom-projected electronic density-of-states (DoS) for the ligand/slab structures, which allowed us to determine the frontier orbital (HOMO and LUMO) energies with respect to the vacuum. Taking the difference of this LUMO with the position of *E*_F_ with respect to the vacuum level for the pristine Ag-(001) slab yielded *E*_b_ at the PBE level of theory. However, these values cannot be considered reliable due to the inaccuracy of semilocal DFT in calculating excited-state properties. Following the DFT+Σ approach described e.g. in ref (28), we applied two corrections to these values. First, we obtained many-body corrections to the HOMO and LUMO of the ligands in the gas phase. Specifically, we obtained the correction to the HOMO as the difference between the Delta self-consistent field (ΔSCF) ionization potential and the position of the PBE HOMO energy with respect to the vacuum. We then followed the procedure proposed in ref (29) and took the LUMO correction to be equal and opposite to the HOMO correction, a method which has been found to be reasonably accurate^45^ and avoids the difficulties associated with computing electron affinities with ΔSCF. Then, we used a classical image-charge expression^43^ to account for the screening response of the Ag to the excited electron, placing the image plane 1 Å above the Ag surface. The full breakdown of quantities and corrections used to construct *E*_b_ is given as Supporting Information.

### Characterization of Ligand Exchanged Silver Nanowires

The characterization of the LE-AgNWs was done using Fourier transform infrared spectroscopy (FTIR), thermogravimetric analysis (TGA), zeta potential, and four point probe measurements.

Attenuated total reflection Fourier transform infrared spectroscopy (Varian Excalibur FTS 3500 FT-IR spectrometer) was performed on AgNW films for wavenumbers between 500 and 4000 cm^–1^. The films were made by drop-casting 0.1 mL of 1 mg/mL methanolic AgNW suspension on a piece of 1.3 cm × 1.3 cm glass substrate. The corresponding methanolic solutions of the pure ligands were also drop-casted on glass slides and characterized for comparison. Each sample was characterized 4 times to ensure that the characteristic peaks detected in the sample are consistent in all measurements.

For TGA measurements (PerkinElmer TG/DTA 6300), approximately 10 mg of each type of dried AgNWs was used. The samples were placed in a crucible into the TGA and heated at 10 °C/min until 550 °C under a nitrogen environment.

The zeta potentials of the AgNW suspensions were characterized using a Malvern Zetasizer Nano ZS. The LE-AgNWs were diluted to 0.01 mg/mL in DI water. The solutions were loaded into a folded capillary cell using a 1 mL syringe. Five zeta potential measurements were performed on each sample, and the average zeta potential value was obtained.

The electrical property of the films was characterized by measuring their sheet resistances using a 4-point probe (Jandel cylindrical probe) with applied currents in the range between 0.1 and 5 μA. Ten measurements were taken at random locations on each film to obtain a representative average sheet resistance.

The optical transmittance of the spin coated AgNW films were characterized using UV–vis spectroscopy (Cary 5000). The transmittance of the films between 250 and 1200 nm were measured with a scan rate of 1 nm/s.

## Supporting Information Available

Additional discussion on analysis related to zeta potential and TGA measurements, additional figures for FTIR spectra, and details related to DFT calculations (PDF)
