# Supplementary material for: Enhancing Conductivity of Silver Nanowire Networks through Surface Engineering Using Bidentate Rigid Ligands
Source: ACS Appl Mater Interfaces. 2024 Jan 10;16(3):4150–9. doi: 10.1021/acsami.3c15207 (PMC10811619; doi:10.1021/acsami.3c15207)
Supplement: Supplementary file 1 — am3c15207_si_001.pdf [file am3c15207_si_001.pdf]

Supporting Information

Enhancing conductivity of silver nanowire networks through surface engineering using bidentate rigid ligands

Wing Chung Liu, Joseph C. A. Prentice, Christopher E. Patrick, Andrew Watt**

Department of Materials, University of Oxford, 16 Parks Road, Oxford OX1 3PH, United Kingdom.

*Corresponding authors: andrew.watt@materials.ox.ac.uk

This supporting information file contains, in order:

- Supplementary Discussion
- Supplementary Figures
- Supplementary Tables
- Supplementary References

Supplementary Discussion

Difference in zeta potential between rigid and flexible ligands

The LE-AgNWs with fumaric acid showed a more negative zeta potential of -32.0 mV compared to -26.7 mV in the succinic acid sample. This can either be explained by the hypothesised flexible and rigid nature of the ligands or simply due to having fewer succinic acid ligands on the AgNWs as shown in the TGA results. From the TGA results for $L = 5$, the relative amount of fumaric acid to succinic acid in their respective LE-AgNW samples was 0.0178:0.0143, which reduces to approximately 1:0.80.

The two different scenarios can be distinguished by considering the mathematical relation between zeta potential and the effective surface charge density, σ_{eff} as described by the Gouy-Chapman equation:[1]

$$\sigma_{eff} = \sqrt{8cN_a\varepsilon_r\varepsilon_0 k_b T}\sinh\left(\frac{q\xi}{2k_bT}\right) \quad \text{(S1)}$$

where c is the ionic concentration in the solution, N_a is the Avogrado's constant, ε_r is the dielectric constant of the solution, ε_0 is the permittivity of vacuum, k_b is the Boltzmann's constant, T is the temperature of the system, q is the electronic charge and ξ is the zeta potential. Since the surface charge density varies linearly with the density of charged groups on the the nanowire surface, Equation S1 can be used with experimental results of the fumaric acid sample to extrapolate the zeta potential of samples with varying amounts of rigid ligands. Scaling down the experimentally obtained σ_{eff} for the fumaric acid sample by 0.8 gave a zeta potential of -29.7 mV. This is still more negative compared to the -26.7 mV observed in the actual succinic acid sample. Therefore, it seems that the lower charge in the succinic acid LE-AgNWs was not just due to having fewer ligands but likely to be due to the flexible nature as hypothesized and illustrated in Figure 3c.

The results from the amine ligands showed a similar observation. The zeta potential of the control AgNW sample at pH 5 is -16.2 mV. The lower magnitude was most likely caused by the presence of additional ions in the solution used to adjust the pH which reduced the magnitude of zeta potentials.[2] For the LE-AgNWs, all samples aside from the aniline had a much more positive zeta potentials due to the presence of unbound positively charged NH_3^+

groups. The observed charge in PDA was much higher than that in DAH, which is a similar observation compared to the acid ligands. In this case, the ratio of PDA to DAH in their respective samples is 1:0.73. Using equation S1 again, scaling down σ_{eff} in the PDA sample by 0.73 yielded a zeta potential of 5.87 mV. This is once again higher than the observed zeta potential of 3.2 mV in the DAH sample. These results support the hypothesis that the geometry of the ligand plays an important role in how the ligands can be attached to the nanowires.

Comparing acid and amine ligands with similar densities of binding groups

From the zeta potential measurements shown in Figure 5a and b, we can identify that fumaric acid LE-AgNWs made with L = 1 and 2.5 show similar zeta potentials as succinic acid LE-AgNWs made with L = 2.5 and 5 respectively. Similar observation can be made between PDA and DAH LE-AgNWs. This allows us to make fair comparisons only between the two acid ligands and two amine ligands. In order to make comparisons across all 4 ligands, we compare the amount of ligands being exchanged onto the AgNWs at these values of L. Using additional TGA results of fumaric acid and PDA LE-AgNWs made with L = 1 and 2.5 (Table S1), we observe that the ligand to silver ratio between fumaric acid and PDA LE-AgNWs were comparable. Therefore, fumaric acid LE-AgNWs should have similar density of binding groups as PDA LE-AgNWs when using the same L during ligand exchange.

Using these information, we can categorise the samples into 2 groups with different densities of binding groups. The high binding group density includes fumaric acid and PDA LE-AgNWs using L = 2.5, and succinic acid and DAP LE-AgNWs using L = 5. Conversely, the low binding group density group consists of fumaric acid and PDA LE-AgNWs using L = 1, and succinic acid and DAP LE-AgNWs using L = 2.5.

Supplementary Figures

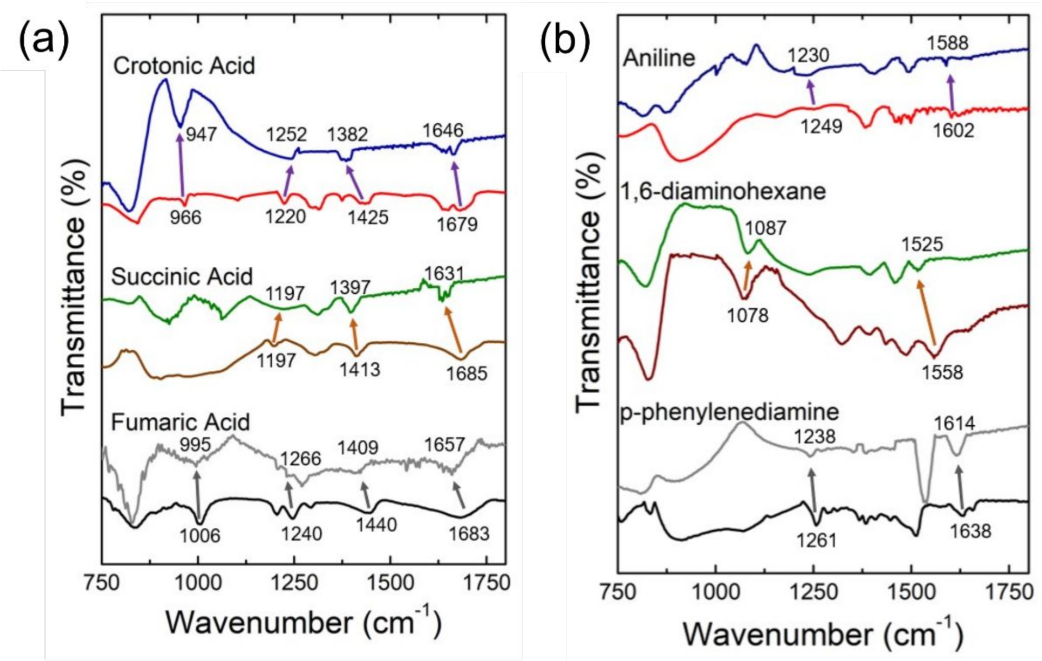

Figure S1: FTIR spectra of: (a) acid ligands and their corresponding LE-AgNWs. (b) Corresponding spectra of amine ligands and their LE-AgNWs. In each pair of spectra, the one below is the spectrum of the pure ligand and the one on top is the spectra of the LE-AgNWs. The shift in characteristic peaks as listed in Table 1 are as labelled.

Supplementary Table

Table S1: TGA results for fumaric acid and PDA LE-AgNWs for L = 1 and 2.5

Ligand	L	Ligand:Ag
Fumaric Acid	1	0.00421
	2.5	0.00934
p-Phenylenediamine	1	0.00398
	2.5	0.00902

Table S2: First-principles-derived quantities used to calculate E_b. All quantities are given in eV.

Ligand	PBE E_b	PBE gas phase HOMO	DSCF gas phase IP	LUMO DSCF correction	LUMO image charge correction	Corrected E_b
Fumaric Acid	0.264	-6.916	9.841	2.925	-0.581	2.608
Succinic Acid	2.205	-6.635	9.527	2.892	-0.655	4.442
Crotonic Acid	1.120	-6.438	9.713	3.275	-0.660	3.734
Aniline	2.284	-4.969	7.727	2.758	-0.542	4.501
p-Phenylenediamine	2.167	-3.833	6.406	2.574	-0.552	4.188
1,6-Diaminohexane	2.708	-5.368	7.764	2.396	-0.603	4.502

Supplementary References

1. Ge, Z. & Wang, Y. Estimation of Nanodiamond Surface Charge Density from Zeta Potential and Molecular Dynamics Simulations. *Journal of Physical Chemistry B* **121**, 3394–3402 (2017).

2. Lowry, G. V. *et al.* Guidance to Improve the Scientific Value of Zeta-Potential Measurements in NanoEHS. *Environ Sci Nano* **3**, 953–965 (2016).
